# First record of *Limnatis paluda* (Hirudinida, Arhynchobdellida, Praobdellidae) from Kazakhstan, with comments on genetic diversity of *Limnatis* leeches

**DOI:** 10.3897/BDJ.3.e5004

**Published:** 2015-04-27

**Authors:** Takafumi Nakano, Tatjana Dujsebayeva, Kanto Nishikawa

**Affiliations:** ‡ Graduate School of Education, Hiroshima University, Higashihiroshima, Japan; § Graduate School of Science, Kyoto University, Kyoto, Japan; | Institute of Zoology MES RK, Almaty, Kazakhstan; ¶ Association for the Conservation of Biodiversity of Kazakhstan (ACBK), Astana, Kazakhstan; # Graduate School of Human and Environmental Studies, Kyoto University, Kyoto, Japan

**Keywords:** Hirudinea, Hirudinida, *Limnatis paluda*, COI, 12S, genetic diversity, geographical record, Kazakhstan

## Abstract

**Background:**

Sawyer (1986) included three species in the nasal leech genus *Limnatis* Moquin-Tandon 1827: *Limnatis nilotica* (Savigny 1822), *Limnatis bacescui* Manoleli 1972 and *Limnatis paluda* (Tennent 1859). The first and last species have mainly been identified in Middle Eastern countries (e.g. Kinzelbach and Rückert 1985). The second species has been identified only in Romania Dobruja (Manoleli 1972). Although *Limnatis* leeches are well known species of endoparasitic leeches, *Limnatis nilotica* was recorded only once in Kazakhstan (Lukin 1976).

**New information:**

Specimens of the genus *Limnatis* from Almaty Province, Kazakhstan are identified as *Limnatis paluda*. This is the first record of *Limnatis paluda* from Kazakhstan. Mitochondrial COI and 12S data demonstrated that the present specimens are genetically close to an Israeli specimen identified as *Limnatis nilotica*. In addition, molecular data suggest that some *Limnatis* specimens whose DNA sequences have been reported were misidentified. According to the observed phylogenetic relationships, the taxonomic status of the known *Limnatis* species should be revisited.

## Introduction

The genus *Limnatis* Moquin-Tandon 1827 is a well known leech taxon of nasal leeches. Leeches of this genus have been reported to infest nasopharyngeal cavities of large mammals, including red deer (Arenas et al. 1993) and humans (Almallah 1968, Boye and Joshi 1994, Harding 1908, Kaburaki 1921, Yakhontov and Ismoilov 1990). Although Soós (1969) placed sixteen species under this genus, Sawyer (1986) grouped only three species in *Limnatis*: *L. nilotica* (Savigny 1822), the type species of the genus, *L. bacescui* Manoleli 1972, and *L. paluda* (Tennent 1859).

The type locality of *L. nilotica* is Egypt, and this species has been reported to occur mainly in Middle Eastern countries (Al-Safadi and El-Shimy 1993, Kinzelbach and Rückert 1985, Nesemann and Forster 1997, Rückert 1985). The second species, *L. bacescui* was only known from its type locality, Romanian Dobruja (Manoleli 1972). As *L. nilotica*, the last species *L. paluda* has mainly been identified in Middle Eastern countries, and is sometimes distributed sympatrically with the first species (Boye and Joshi 1994, Kinzelbach and Rückert 1985, Kuntz and Myers 1968, Rückert 1985). However, *Limnatis paluda* was first described from Ceylon (Sri Lanka) and was stated to be a cattle leech there (Tennent 1859). Its taxonomic status was revisited by Moore (1927a). Although Moore could not examine Ceylonese materials of *L. paluda*, many studies followed his classification on this species. Despite its impact as a parasite of humans and other large mammals, there have been very few distributional studies of Kazakhstan nasal leeches of *Limnatis*. To our knowledge, *L. nilotica* was identified from Bilikol Lake, Jambyl Province, southern Kazakhstan by Lukin (1976). In addition, this species was recently recorded in Kazakhstan along with neighboring countries, Azerbaijan and Uzbekistan (Kovalenko and Utevsky 2015). The second (TD) and last (KN) authors collected *Limnatis* leeches from Almaty Province, southeastern Kazakhstan, near the border with China. Based on morphological examination and molecular phylogenetic analyses, the taxonomic status of this leech was clarified. In addition, its genetic diversity based on mitochondrial DNA data is briefly discussed.

## Materials and methods

**Figure 1.**
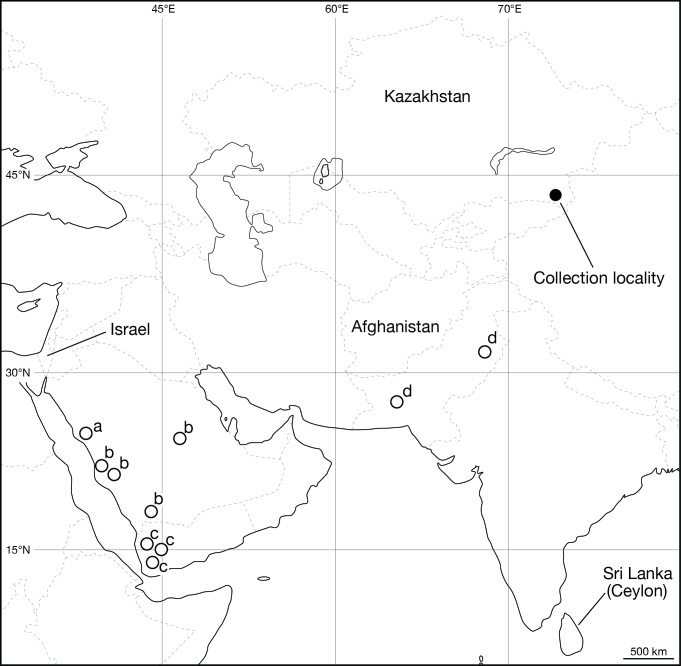
Localities where *Limnatis paluda* (Tennent 1859) has been recorded. Open circles indicate collection localities in previous studies. Sources: ^a^Boye and Joshi (1994); ^b^Kinzelbach and Rückert (1985); ^c^Kuntz and Myers (1968); ^d^Moore (1927a).

Leeches were collected from the Suygaty Valley located at the left bank part of the Ily River Depression, Almaty Province, Kazakhstan (Fig. 1). The specimens were preserved in absolute ethanol (EtOH) in the field. For DNA extraction, botryoidal tissue was removed from the posterior part of the body around the caudal sucker of every specimen, and then preserved in absolute EtOH. The remainder of the body was fixed in 10% formalin and preserved in 70% EtOH. Four measurements were taken: body length (BL) from the anterior margin of the oral sucker to the posterior margin of the caudal sucker, maximum body width (BW), caudal sucker length (CL) from the anterior to the posterior margin of the caudal sucker and caudal sucker width (CW) from the right margin to the left margin of the sucker. Examination, dissection of 2 specimens (KUZ Z702 and Z703; see below), and drawing of the specimens were conducted using a stereoscopic microscope with a drawing tube (Leica M125). Specimens used in this study have been deposited in the Zoological Collection of Kyoto University (KUZ).

The numbering convention is based on Moore (1927b): body somites are denoted by Roman numerals and the annuli in each somite are given alphanumeric designations.

Sequences of mitochondrial COI as well as 12S, tRNA^Val^ and 16S (12S–16S) were determined for 2 specimens from Almaty Province. The extraction of genomic DNA and DNA sequencing methods followed Nakano (2012). Primer sets for the PCR and cycle sequencing (CS) reactions used in this study were as follows: for COI, LCO 1490 (PCR and CS) and HCO 2198 (CS) (Folmer et al. 1994), and LCO-in2 (CS) (5’-GCTATTACAATATTACTTACAGATCG-3’) (this study) and HCO-out (PCR and CS) (Nakano 2012); for 12S–16S, 12SA-in (PCR and CS) and 12SB-out (PCR and CS) (Nakano 2012). The PCR reaction mixtures were heated to 94°C for 5 min, followed by 40 cycles at 94°C (10 s each), 43°C for COI or 52°C for 12S–16S (20 s), and 72°C (42 s each), and a final extension at 72°C for 6 min. The sequencing mixtures were heated to 96°C for 2 min, followed by 40 cycles at 96°C (10 s each), 50°C (5 s each), and 60°C (42 s each). The obtained sequences were edited using DNA BASER (Heracle Biosoft S.R.L.). The DNA sequences listed in Table 1 were newly obtained in this study, and were deposited with the International Nucleotide Sequence Database Collaboration (INSDC).

To determine the phylogenetic position of Kazakhstani *Limnatis*, 10 previously published sequences were obtained from the INSDC for use in molecular phylogenetic analyses (Table 1). One praobdellid species, *Limnobdella mexicana* Blanchard 1893, which was identified as a sister lineage in previous phylogenetic studies (Phillips et al. 2010, Phillips and Siddall 2009), was used as an outgroup taxon.

Sequences of mitochondrial COI were aligned by eye, as no indels were observed. Mitochondrial 12S–16S sequences were aligned using MAFFT v. 7.213 FFT-NS-2 (Katoh and Standley 2013). Genetic distances for each marker were calculated by an uncorrected p-distance, and then neighbour-joining trees were constructed with nonparametric bootstrapping (Felsenstein 1985) based on 1,000 replicates using MEGA 6 (Tamura et al. 2013). All missing positions were eliminated for each sequence pair. Therefore, the tRNA^Val^ and 16S regions were removed from the 12S–16S sequences, when uncorrected p-distances were calculated.

## Taxon treatments

### Limnatis paluda

(Tennent, 1859)

#### Materials

- **Type status:** Other material. **Occurrence:** catalogNumber: Z700; recordedBy: Kanto Nishikawa; individualCount: 1; sex: hermaphrodite; **Taxon:** scientificName: *Limnatis paluda* (Tennent, 1859); **Location:** country: Kazakhstan; stateProvince: Almaty; verbatimLocality: Suygaty Valley, Almaty Province, Kazakhstan; decimalLatitude: 43.512778; decimalLongitude: 78.9708331; **Identification:** identifiedBy: Takafumi Nakano; **Event:** eventDate: 2013-06-21; **Record Level:** institutionCode: KUZ
- **Type status:** Other material. **Occurrence:** catalogNumber: Z701; recordedBy: Kanto Nishikawa; individualCount: 1; sex: hermaphrodite; **Taxon:** scientificName: *Limnatis paluda* (Tennent, 1859); **Location:** country: Kazakhstan; stateProvince: Almaty; verbatimLocality: Suygaty Valley, Almaty Province, Kazakhstan; decimalLatitude: 43.512778; decimalLongitude: 78.9708331; **Identification:** identifiedBy: Takafumi Nakano; **Event:** eventDate: 2013-06-21; **Record Level:** institutionCode: KUZ
- **Type status:** Other material. **Occurrence:** catalogNumber: Z702; recordedBy: Kanto Nishikawa; individualCount: 1; sex: hermaphrodite; **Taxon:** scientificName: *Limnatis paluda* (Tennent, 1859); **Location:** country: Kazakhstan; stateProvince: Almaty; verbatimLocality: Suygaty Valley, Almaty Province, Kazakhstan; decimalLatitude: 43.512778; decimalLongitude: 78.9708331; **Identification:** identifiedBy: Takafumi Nakano; **Event:** eventDate: 2013-06-21; **Record Level:** institutionCode: KUZ
- **Type status:** Other material. **Occurrence:** catalogNumber: Z703; recordedBy: Kanto Nishikawa; individualCount: 1; sex: hermaphrodite; **Taxon:** scientificName: *Limnatis paluda* (Tennent, 1859); **Location:** country: Kazakhstan; stateProvince: Almaty; verbatimLocality: Suygaty Valley, Almaty Province, Kazakhstan; decimalLatitude: 43.512778; decimalLongitude: 78.9708331; **Identification:** identifiedBy: Takafumi Nakano; **Event:** eventDate: 2013-06-21; **Record Level:** institutionCode: KUZ

#### Description

Body firm, muscular, with constant width in caudal direction, dorsoventrally compressed, BL 22.64–36.73 mm, BW 4.47–9.82 mm (Fig. 2a). Caudal sucker elliptic, CL 3.94–6.26 mm, CW 4.11–7.17 mm (Fig. 2b).

##### Annulation

**Figure 4.**
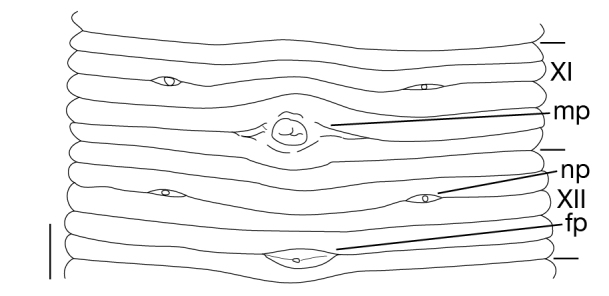
*Limnatis paluda* (Tennent 1859) from Suygaty Valley, Kazakhstan, KUZ Z702. Ventral view of somites XI and XII. Abbreviations: fp, female gonopore; mp, male gonopore; np, nephridiopore. Scale bar: 0.5 mm.

Somite I completely merged with prostomium (Fig. 3a). somites II and III unite together, forming 1 annulus (Fig. 3a, b). Somite IV biannulate, (a1 + a2) > a3; in KUZ Z703, (a1 + a2) with slight dorsal furrow (Fig. 3a, b). Somite V biannulate, (a1 + a2) = a3 (Fig. 3a, b); in KUZ Z700 and Z703, (a1 + a2) with slight dorsal furrow. IV a3–V a3 unite altogether, forming posterior margin of oral sucker (Fig. 3c). Somite VI dorsally triannulate/ventrally biannulate, a1 = a2 = a3/(a1 + a2) > a3 (Fig. 3). Somite VII triannulate, a1 = a2 = a3 (Fig. 3). Somite VIII quadrannulate, a1 = a2 = b5 = b6 (Fig. 3). Somites IX–XXIII quinquannulate, b1 = b2 = a2 = b5 = b6 (Figs 4, 5). Somite XXIV quadrannulate, b1 = b2 = a2 = a3 (Fig. 5a). Somite XXV triannulate, a1 = a2 = a3 (Fig. 5a); XXV a1 (KUZ Z702), a2 (KUZ Z703), or a3 (KUZ Z700 and Z701) being last complete annulus on venter. Somite XXVI biannulate, (a1 + a2) = or > a3 (Fig. 5a); in KUZ Z701 and Z703, (a1 + a2) with slight dorsal furrow. Somite XXVII uni- (KUZ Z702) or biannulate (Fig. 5a). Anus at last annulus of XXVII (KUZ Z700) or behind XXVII (Fig. 5a).

##### Gonopores

Male gonopore in XI b5/b6 (Fig. 4). Female gonopore in XII b5/b6 (Fig. 4). Gonopores separated by 5 annuli.

##### Sense organs

Eyes 5 pairs, in parabolic arc; 1st and 2nd pairs on II + III, 3rd pair on IV (a1 + a2), 4th pair on V (a1 + a2), and 5th pair on VI a2 (Fig. 3a, b). Sensillae, papillae undeveloped.

##### Nephridiopores

In 17 pairs, one each situated ventrally at posterior margin of VIII a1 and b2 of each somite in IX–XXIV (Figs 3c, 4, 5b).

##### Digestive tract

1 median longitudinal furrow on ventral surface of oral sucker (Fig. 3b). 3 jaws situated in oral cavity, one dorsal and two ventrolateral, each jaw bearing numerous salivary papillae. Monostichodont: each jaw bearing 30–46 diminutive teeth. Pharynx reaching to IX b2/a2–a2/b5. Crop reaching to XX b2/a2, bearing 22 pairs of crop caeca: 1st pair IX; 2nd and 3rd in IX and X; 4th and 5th in X and XI, 4th larger than 5th (4th > 5th); 6th and 7th in XI and XII, 6th > 7th; 8th and 9th in XII and XIII, 8th > 9th; 10th and 11th in XIII and XIV, 10th > 11th; 12th and 13th in XIV and XV, 12th > 13th; 14th and 15th in XV and XVI, 14th > 15th; 16th and 17th in XVI and XVII, 16th > 17th; 18th and 19th in XVII and XVIII, 18th > 19th; 20th and 21st in XVIII and XIX, 20th > 21st; 22nd being post-crop caeca, in XIX b2–a2 to XXV a2–a3. Intestine reaching to XXII/XXIII. Rectum simple tubular.

##### Male genital organ

**Figure 6.**
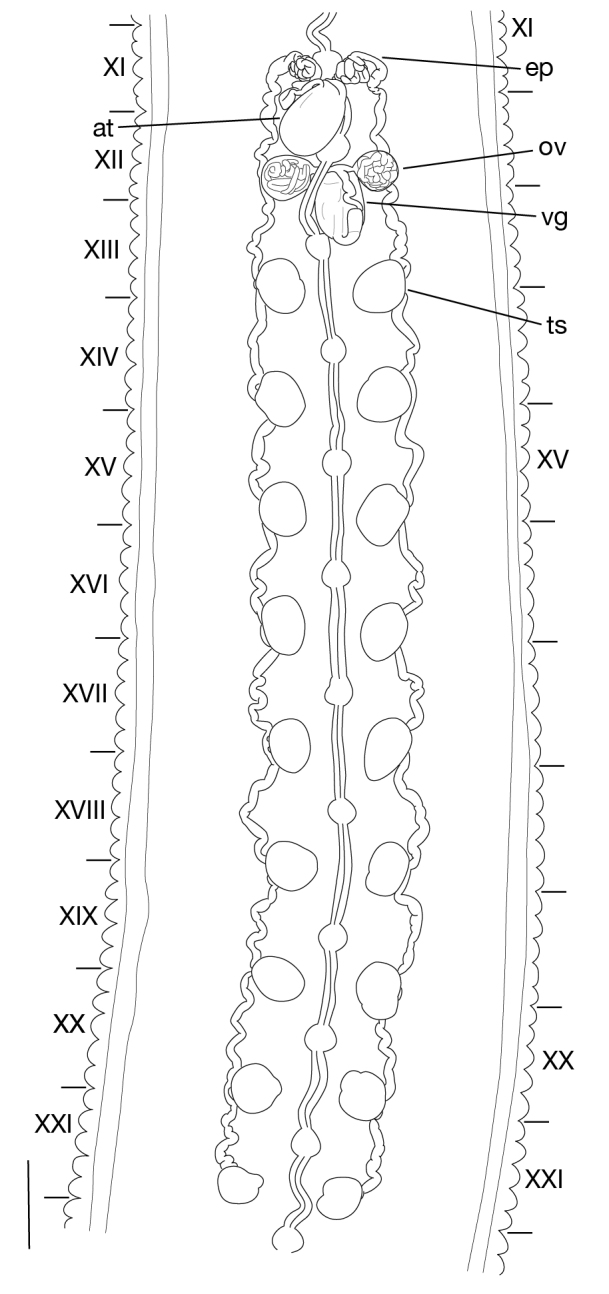
*Limnatis paluda* (Tennent 1859) from Suygaty Valley, Kazakhstan, KUZ Z702. Dorsal view of reproductive system including ventral nervous system. Abbreviations: at, atrium; ep, epididymis; ov, ovisac; ts, testisac; vg, vagina. Scale bar: 1 mm.

Testisacs 8 or 9 pairs (Fig. 6): 1st pair in XIII b5–XIV b1; 2nd pair in XIV b5–XV b1; 3rd pair in XV b5–XVI b1; 4th pair in XVI b5–XVII b1; 5th pair in XVII b5–XVIII b1; 6th pair in XVIII a2–XIX b1; 7th pair in XIX b5–XX b1; 8th pair in XX b5–XXI b1; 9th pair in XXI b5–XXII b1. Paired epididymides small, globular; right epididymis in XI a2–XII b2, left epididymis in XI a2–XII b1 (Figs 6, 7). Ejaculatory bulb absent. Ejaculatory ducts folded reaching to anterior end of male atrium (Fig. 7). Atrium continuous with penis sheet (Fig. 7). Penis sheet reaching to XII a2–b2, then turning anteriorly to male gonopore, U-shaped (Figs 6, 7).

##### Female genital organ

Pair of ovisacs globular, in XII b5–XIII b1 (Figs 6, 8). Oviducts short, borth oviducts converging into common oviduct in XII b5–XIII b1 (Fig. 8). Common oviduct slightly folded, descending to female vagina (Fig. 8). Vagina ellipsoid, straight, directly descending to female gonopore (Fig. 8).

##### Colour

**Figure 9.**
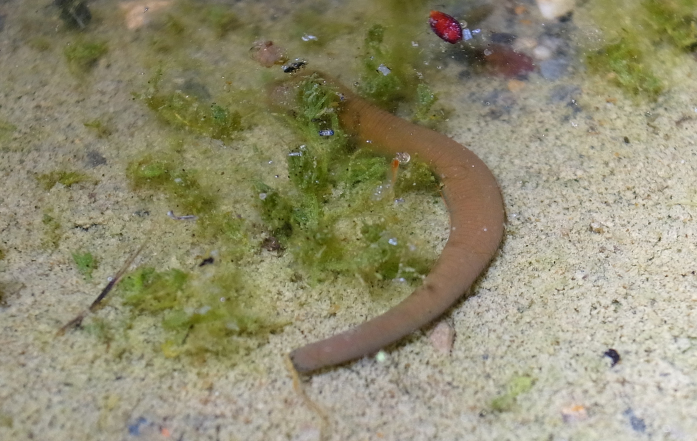
*Limnatis paluda* (Tennent 1859) from Suygaty Valley, Kazakhstan. Dorsal view of a live animal, one of the leeches examined in this study, in the field. Photo taken by KN.

When alive, dorsal surface uniform reddish brown (Fig. 9); ventral surface paler than dorsal surface; both lateral marginal stripes orange in VI a3 to XXVI (a1 + a2)–a3. In preservative, colour slightly faded (Fig. 2).

#### Habitat

During night time, the leeches examined in this study were found crawling in a small pond (Figs 9, 10a) fed from an artificial well on a small hill. The hill is situated in the clayey gravelly desert at the foot of the arid low mountains Ulken Boguty at 1270 a.s.l. (Fig. 10b). Although at one time local inhabitants grazed domesticated animals around the well, at present there are no dwellers in the area.

#### Genetic data

**Figure 11.**
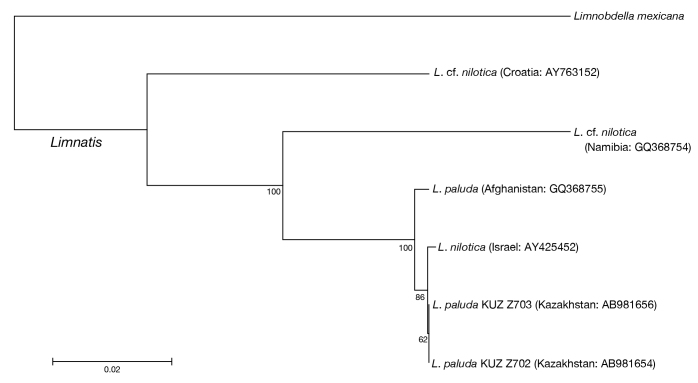
The neighbour-joining tree of available COI sequences. Numbers associated with nodes represent bootstrap values. Length of each sequence is listed in Table 1.

**Figure 12.**
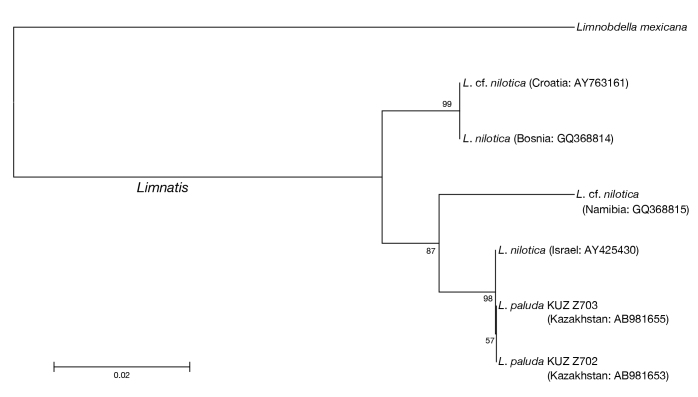
The neighbour-joining tree of available 12S sequences. Numbers associated with nodes represent bootstrap values. Length of each sequence is listed in Table 1.

Neighbour-joining trees generated based on both the COI (Fig. 11) and 12S (Fig. 12) genes showed that the present *Limnatis* specimens from Kazakhstan form a monophyletic lineage with a sequence from *L. nilotica* collected in Israel (BS = 86% in COI, 98% in 12S). In the neighbour-joining tree based on COI sequences, this monophyletic lineage and one sequence obtained from the Afghan *L. paluda* formed a well-recovered clade (BS = 100%). In addition, the neighbour-joining trees revealed that sequences obtained from *L. nilotica* collected in Namibia and Croatia do not form a monophyletic group.

The COI uncorrected p-distance between the Kazakhstani *L. paluda* and the Israeli *L. nilotica* was 0.2% (Table 2) based on 12S sequences consisting of 353 bp which showed that the sequences of the former are identical with that of the latter (Table 3). The COI uncorrected p-distance between Kazakhstani *L. paluda* and that from Afghanistan was 0.5%, and that between the Israeli *L. nilotica* and the Afghan *L. paluda* was 0.6%. The COI and 12S uncorrected p-distances between the Kazakhstani, Israeli, and Afghan *Limnatis* sequences and the remaining sequences of *L. nilotica* were 7.3–9.7% and 2.8%, respectively. The COI uncorrected p-distance between the Namibian L. cf. nilotica and the Croatian L. cf. nilotica was 11.9%, and that based on 12S was 3.9%.

#### Taxon discussion

The present 4 specimens of *Limnatis* clearly belong to *Limnatis paluda* sensu Moore (1927a) based on the following characteristics: VI a1 and a2 forming 1 annulus (a1 + a2) on venter; XXIV quadrannulate; sensillae small, undeveloped; each jaw bearing numerous salivary papillae; monostichodont teeth numbering 30–46 on each jaw; and body surface uniform brownish with lateral marginal stripes in orange. Tennent (1859) provided incomplete morphological characteristics of *L. paluda*, describing its colour as uniform brown without any bands but with reddish lateral margins, and not very numerous teeth. Although Moore (1927a) could not examine specimens of *L. paluda* from its type locality, he identified *Limnatis* leeches from Punjab and Baluchistan, which presently belong to Pakistan, as *L. paluda* based on their colouration as well as their limited number of teeth on each jaw.

Moore (1927a) stated that *L. paluda* could be clearly distinguished from *Limnatis nilotica* by its limited number (30–47) of teeth on each jaw (the latter species has numerous teeth on each jaw). He also mentioned that the numbers of teeth in *L. paluda* and *L. nilotica* never overlapped. Kinzelbach and Rückert (1985) also mentioned that the number of teeth in *L. paluda* was at most 45, but that of *L. nilotica* ranged from 45 to 60. However, Lukin (1962) and Lukin (1976) noted that *L. nilotica* possessed 30–50 teeth on each jaw. Based on the specimens collected in Azerbaijam, Kazakhstan and Uzbekistan, Kovalenko and Utevsky (2015) reported that *L. nilotica* bore 38–40 teeth on each jaw. In addition, *L. bacescui* possessed 50–54 teeth on each jaw (Manoleli 1972). Although *Limnatis* species are well known as nasal leeches, the taxonomic status of each species seems to be far from clarified. Each nominal species of the genus *Limnatis* including *L. paluda* should be revised based on specimens collected from the type locality.

As mentioned above, the taxonomic identities of *Limnatis* species have not been fully settled. According to the present neighbour-joining trees and p-distance data, however, the Israeli *Limnatis* leech, of which DNA sequences have been deposited with INSDC, should be assigned to *Limnatis paluda* as mentioned in (Phillips and Siddall 2009), because it forms a monophyly with the present Kazakhstani *L. paluda* specimens, and the p-distances of the COI and 12S sequences show extremely low genetic divergence (0.2% in COI and no differences in 12S). In addition, it is highly likely that L. cf. nilotica of Croatia and Bosnia and L. cf. nilotica of Namibia do not belong to the same species, because those specimens are paraphyletic consistently in the present phylogenetic trees, and the former is highly diverged from the latter (11.9% in COI and 3.9% in 12S). These uncorrected p-distance values are greater than those between *L. paluda* and the Namibian *Limnatis* species (7.3–7.4% in COI and 2.8% in 12S) as well as *L. paluda* and the Balkan *Limnatis* specimens (9.2–9.7% in COI and 2.8% in 12S).

It is noteworthy that the specimens of *Limnatis paluda* analysed in this study have low genetic divergences (0.2–0.5% in COI and 0% in 12S). The COI uncorrected p-distances between the present Kazakhstani specimens and the Israeli specimen are lower than that between the former and the Afghan specimen (0.5%) and that between the latter and the Afghan *L. paluda* (0.6%). The collection locality in Kazakhstan is ca. 4,000 km from Israel, and ca. 2,000 km from Afghanistan. In contrast, Israel is at most ca. 3,500 km from Afghanistan. Because few DNA sequences of *L. paluda* are available, it may be difficult to reveal its detailed genetic structure. However, the results of the mitochondrial genetic analyses at least shed light on the discordance between the COI genetic divergence between the Kazakhstani *L. paluda* and the Israeli specimen and the geographic distance between the collection localities. Trontelj and Utevsky (2012) revealed low genetic diversity in the European medicinal leeches, and suggested that medicinal leeches dispersed rapidly and widely via their host animals as these leeches are ectoparasitic species. Because *Limnatis* species are endoparasitic leeches that attach to the nasopharyngeal cavities of mammals, they likely achieve long-distance dispersal. Except when they parasitise the mammalian nasopharyngeal cavities, *Limnatis* species generally inhabit a freshwater environment. One of the routes of the Silk Road passed through the Ili River Depression (Baipakov 1998), near the collection locality of the present specimens. Therefore, *Limnatis paluda* have possibly dispersed by means of freshwater places along the trade route as stepping stones, and thus human activities may have influenced the distribution of *L. paluda*. In either case, further taxonomic studies of *Limnatis* species should be performed to clarify their taxonomic positions. In addition, future molecular studies should elucidate the biogeographical history of *Limnatis paluda*.

## Supplementary Material

XML Treatment for Limnatis paluda

## Figures and Tables

**Figure 2a.**
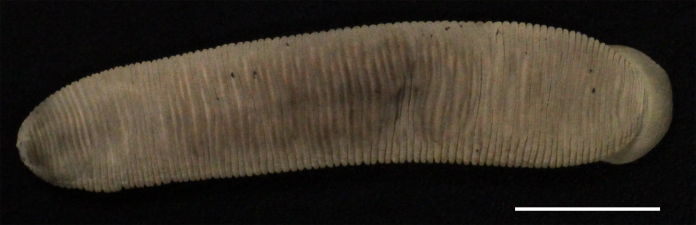
dorsal view.

**Figure 2b.**
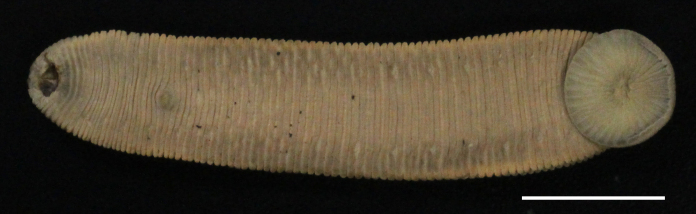
ventral view.

**Figure 3a.**
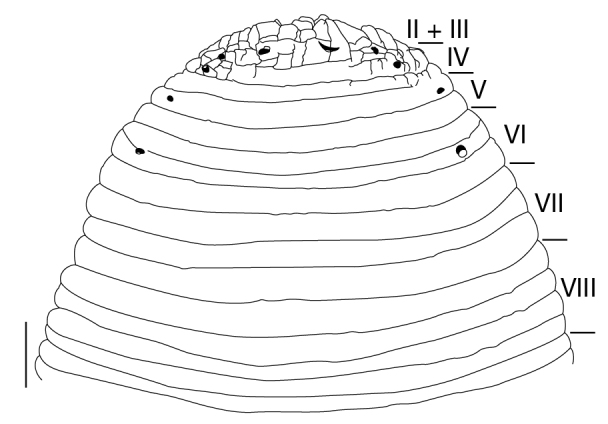
dorsal view of somites I–VIII.

**Figure 3b.**
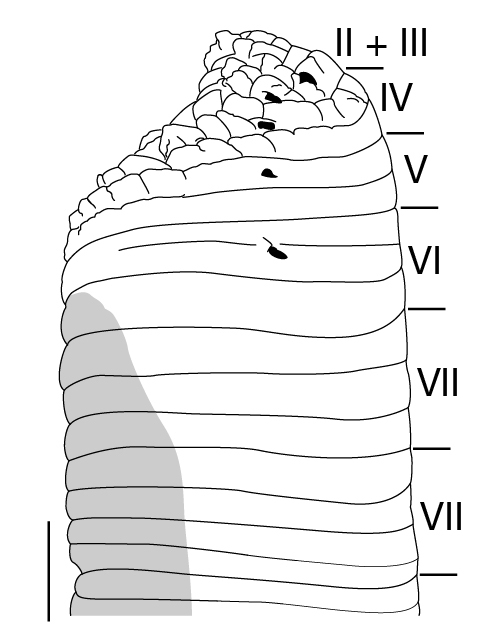
left lateral view of somites I–VIII.

**Figure 3c.**
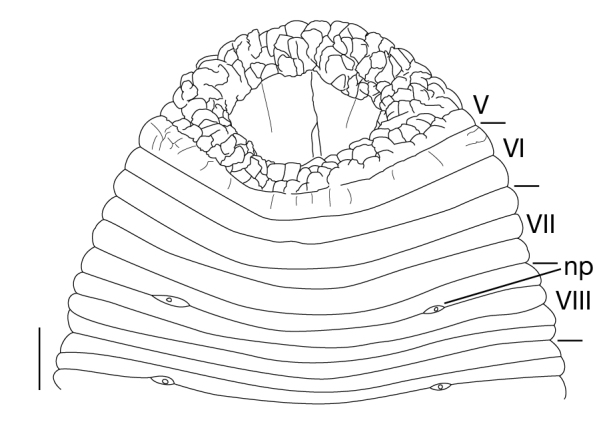
ventral view of somites I–VIII.

**Figure 5a.**
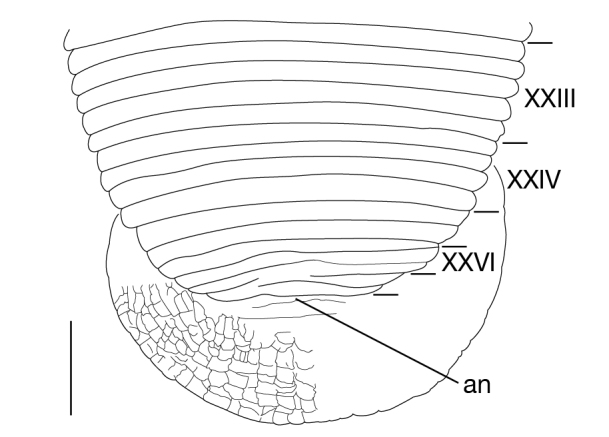
dorsal view of somites XXIII–XXVII and caudal sucker.

**Figure 5b.**
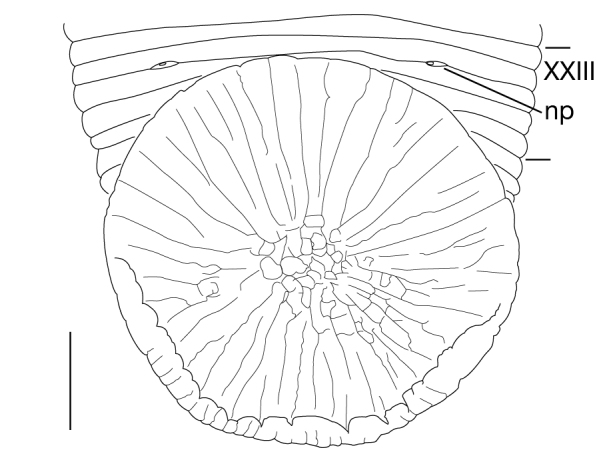
ventral view of somites XXIII and caudal sucker.

**Figure 7a.**
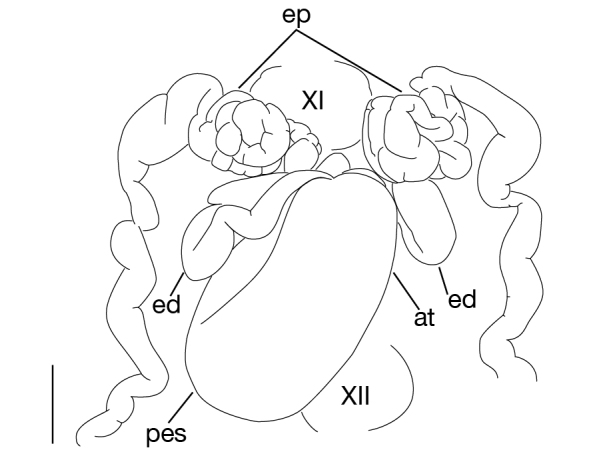
dorsal view of male median reproductive system including positions of ganglia XI and XII. Scale bar: 0.25 mm.

**Figure 7b.**
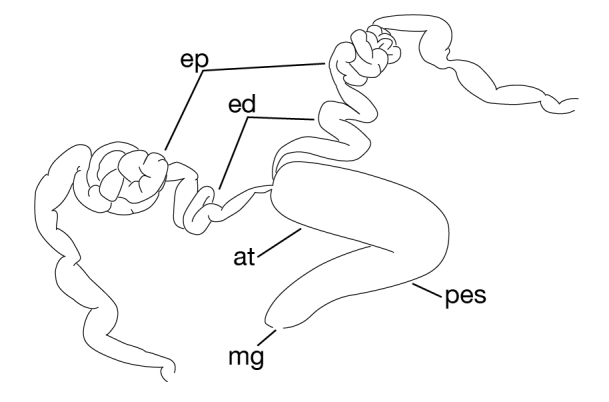
left lateral view of schematic drawing of male median reproductive system.

**Figure 8a.**
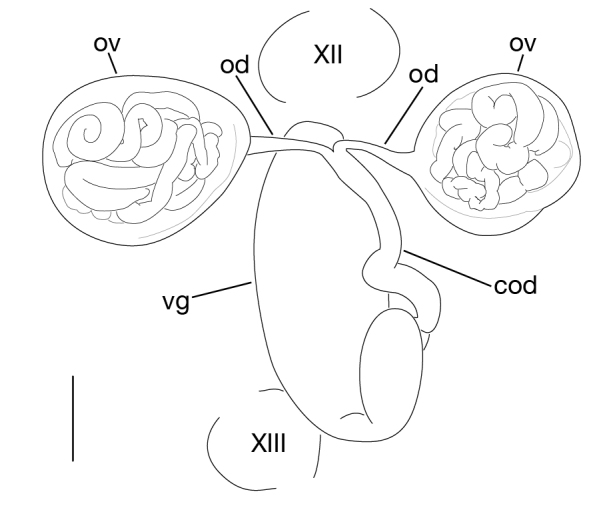
dorsal view of female reproductive system including positions of ganglia XII and XIII. Scale bar: 0.25 mm.

**Figure 8b.**
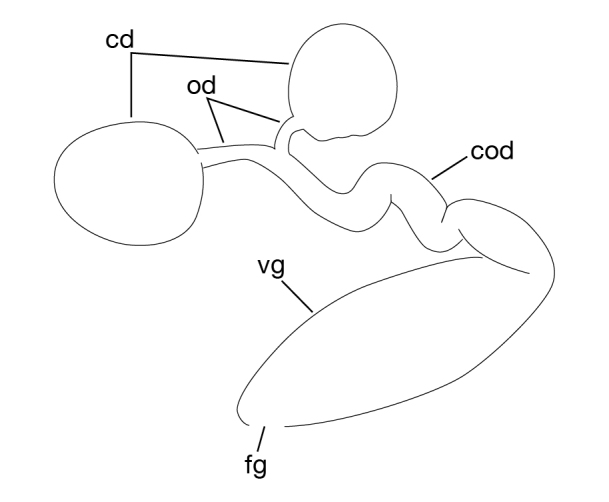
left lateral view of schematic drawing of female reproductive system.

**Figure 10a.**
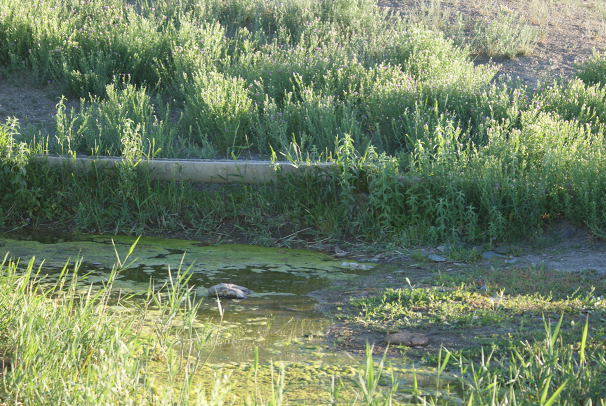
small pond where the present leeches were found. Photo taken by Dr Atsushi Tominaga.

**Figure 10b.**
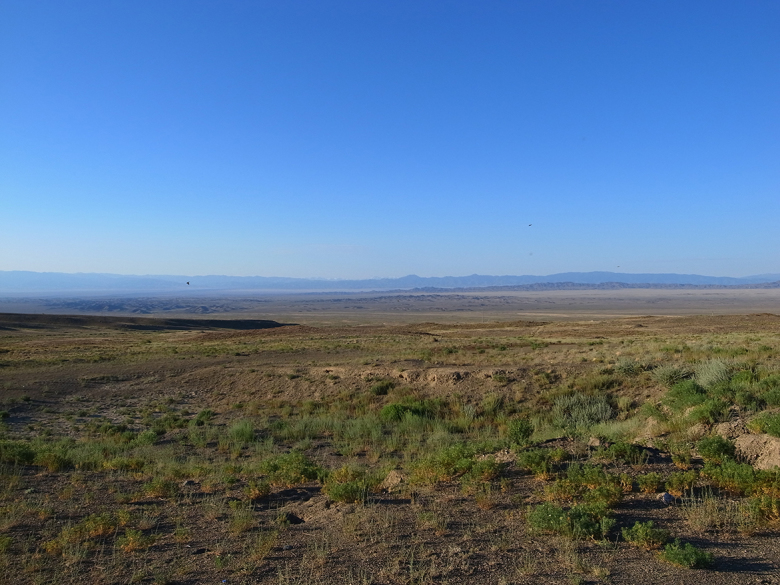
landscape of the Suygaty Valley (Ili River Depression). Photo taken by KN.

**Table 1.** Samples with voucher or isolate numbers, collection country and INSDC accession numbers used for molecular analyses. Sequences marked with an asterisk (*) were obtained for the first time in the present study. Acronym: KUZ, Zoological Collection of Kyoto University. Sources: ^a^Borda and Siddall (2004); ^b^Trontelj and Utevsky (2005).

Species (voucher or isolate #)	Country	COI		12S–16S or 12S	
		Accession #	Length	Accession #	Length
*Limnatis paluda* (KUZ Z702)	Kazakhstan	AB981654 *	1267	AB981653 *	601
*Limnatis paluda* (KUZ Z703)	Kazakhstan	AB981656 *	1267	AB981655 *	601
*Limnatis paluda* (AFLP)	Afghanistan	GQ368755	658		
*Limnatis nilotica*	Israel^a^	AY425452	648	AY425430	353
Limnatis cf. nilotica (AOBS)	Namibia	GQ368754	565	GQ368815	355
*Limnatis nilotica*	Bosnia			GQ368814	355
Limnatis cf. nilotica	Croatia^b^	AY763152	631	AY763161	503
*Limnobdella mexicana* (LM117)	Mexico	GQ368758	658	GQ368818	353

**Table 2.** Uncorrected p-distances for available COI sequences of *Limnatis* leeches. Length of each sequence is listed in Table 1. Acronym: KUZ, Zoological Collection of Kyoto University.

Species	Country	1	2	3	4	5	6
1. *Limnatis paluda* (KUZ Z702)	Kazakhstan						
2. *Limnatis paluda* (KUZ Z703)	Kazakhstan	0.000					
3. *Limnatis nilotica*	Israel	0.002	0.002				
4. *Limnatis paluda*	Afghanistan	0.005	0.005	0.006			
5. Limnatis cf. nilotica	Namibia	0.073	0.073	0.073	0.074		
6. Limnatis cf. nilotica	Croatia	0.095	0.095	0.097	0.092	0.119	

**Table 3.** Uncorrected p-distances for available 12S sequences of *Limnatis* leeches. Length of each sequence is listed in Table 1. Acronym: KUZ, Zoological Collection of Kyoto University.

Species	Country	1	2	3	4	5	6
1. *Limnatis paluda* (KUZ Z702)	Kazakhstan						
2. *Limnatis paluda* (KUZ Z703)	Kazakhstan	0.000					
3. *Limnatis nilotica*	Israel	0.000	0.000				
4. Limnatis cf. nilotica	Namibia	0.028	0.028	0.028			
5. *Limnatis nilotica*	Bosnia	0.028	0.028	0.028	0.039		
6. Limnatis cf. nilotica	Croatia	0.028	0.028	0.028	0.039	0.000	
